# +3179G/A Insulin-Like Growth Factor-1 Receptor Polymorphism: A Novel Susceptibility Contributor in Anti-Ro/SSA Positive Patients with Sjögren’s Syndrome: Potential Clinical and Pathogenetic Implications

**DOI:** 10.3390/jcm10173960

**Published:** 2021-08-31

**Authors:** Charalampos Skarlis, Nikolaos Marketos, Adrianos Nezos, Asimina Papanikolaou, Michael Voulgarelis, Michael Koutsilieris, Haralampos M. Moutsopoulos, Clio P. Mavragani

**Affiliations:** 1 Department of Physiology, School of Medicine, National and Kapodistrian University of Athens, 11527 Athens, Greece; charskarlis@med.uoa.gr (C.S.); nickmarketos@yahoo.com (N.M.); anezos@med.uoa.gr (A.N.); mkoutsil@med.uoa.gr (M.K.); 2 Department of Hematopathology, Evangelismos Hospital, 11527 Athens, Greece; aspapanikolaou@yahoo.com; 3 Department of Pathophysiology, School of Medicine, National and Kapodistrian University of Athens, 11527 Athens, Greece; mvoulgar@med.uoa.gr (M.V.); hmoutsop@med.uoa.gr (H.M.M.); 4 Medical Sciences/Immunology, Academy of Athens, 11527 Athens, Greece; 5 Joint Academic Rheumatology Program, School of Medicine, National and Kapodistrian University of Athens, 11527 Athens, Greece

**Keywords:** Sjögren’s syndrome, insulin growth factor receptor, rs2229765, anti-Ro/SSA, autoimmunity, pyroptosis

## Abstract

Background: Alterations of the insulin-like growth factor (IGF) pathway along with genetic variations of the IGF1 receptor (IGF1R) gene have been linked to the development of systemic autoimmunity, possibly through apoptosis induction. This study aims to investigate whether genetic variations of the IGF1R contribute to Sjögren’s syndrome (SS) pathogenesis and explores potential functional implications. Methods: DNA extracted from whole peripheral blood derived from 277 primary SS patients, complicated or not by lymphoma, and 337 Healthy controls (HC) was genotyped for the rs2229765 IGF1R polymorphism using the RFLP-PCR assay. Gene expression of IGF1R and IGF1 isoforms, caspases 1, 4, and 5, and inflammasome components NLRP3, ASC, IL1β, IL18, IL33, IGFBP3, and IGFBP6 were quantitated by RT-PCR in total RNA extracted from minor salivary gland biopsies (MSGs) of 50 SS patients and 13 sicca controls (SCs). In addition, IGF1R immunohistochemical (IHC) expression was assessed in formalin-fixed, paraffin-embedded MSG tissue sections derived from 10 SS patients and 5 SCs. Results: The prevalence of the A/A genotype of the rs2229765 IGF1R polymorphism was significantly higher in the anti-Ro/SSA positive SS population compared to healthy controls (24.8% vs. 10.7%, *p* = 0.001). Moreover, IGF1Rs at both mRNA and protein levels were reduced in SS-derived MSGs compared to SCs and were negatively associated with caspase 1 transcripts. The latter were positively correlated with NLRP3, ASC, and IL1β at the salivary gland tissue level. IGF1R expression in peripheral blood was negatively correlated with ESR and IgG serum levels and positively correlated with urine-specific gravity values. Conclusions: The rs2229765 IGF1R variant confers increased susceptibility for seropositive primary SS. Dampened IGF1R mRNA and protein expression in salivary gland tissues could be related to increased apoptosis and subsequently to the activation of inflammasome pathways.

## 1. Introduction

Primary Sjögren’s syndrome (SS) is a chronic autoimmune disease of indolent course mainly characterized by oral and ocular dryness resulting from lymphocytic infiltration in salivary and lacrimal glands, respectively. Given that other organs may also be affected, including joints, lungs, kidneys, liver, and nervous system, primary SS is considered a systemic disease [1,2], affecting mainly perimenopausal women with a female-to-male predominance about 9:1 [3]. Disease hallmarks include B-cell hyperactivity, oversecretion of serum autoantibodies such as anti-Ro/SSA, anti-La/SSB, and rheumatoid factor (RF), as well as the activation of type I and type II interferon (IFN) pathways [4,5,6,7]. The most severe disease complication is the development of non-Hodgkin’s lymphoma (NHL), which occurs in 5–10% of primary SS patients and represents the leading cause of disease-related mortality [8,9,10].

Though the precise etiology of primary SS remains largely unexplored, the interplay between distinct genetic background, environmental triggers, as well as endocrine alterations have been recognized as significant contributors in SS pathogenesis leading to salivary gland dysfunction [11,12,13].

Programmed cell death (PCD) dysregulation is considered a pivotal etiopathogenetic mechanism for SS development [14,15]. Particularly, increased apoptosis of salivary gland epithelial cells (SGECs) along with impaired clearance of apoptotic debris lead to augmented immunocomplex formation, which further activates the local immune response, contributing to salivary gland dysfunction [15,16]. Of note, previous studies have suggested that the NLR family pyrin domain containing 3 (NLRP3)-inflammasome-mediated inflammatory PCD (named pyroptosis) plays a crucial role in both SS pathogenesis and SS-related lymphomagenesis [17,18,19,20]. However, the initial triggers leading to inflammasome activation are still unknown.

The insulin-like growth factor (IGF) pathway includes insulin-like growth factors (IGF1 and IGF2), insulin-like growth factor binding proteins (IGFBPs 1–6), and insulin-like growth factor receptors (IGF1R and IGF2R) [21,22,23]. IGF1R is the primary signal transducer of the proliferative and anti-apoptotic activity of IGF1, maintaining cellular homeostasis. The detection of IGF1 in both human and animal model salivary gland epithelial cells implies a potential role of the IGF pathway in salivary gland function [24]. Notably, dysregulation of the IGF pathway has been observed in the setting of chronic autoimmune/inflammatory diseases, suggesting a potential contributory role in autoimmune pathogenesis [25]. In the SS context, reduced immunohistochemical detection of IGF1R has been previously shown in an animal model of experimental autoimmune sialadenitis [26] as well as in salivary gland tissues from SS patients [27], along with increased IGF1 expression [28], compared to controls. Furthermore, microarray analysis on peripheral blood monocytes showed decreased expression of IGF1R transcripts in SS patients [29]. Additionally, IGF1R polymorphism rs2229765 (a synonymous transition +3179G>A in exon 16, codon 1013)—particularly the A/A genotype and A allele—has been previously shown to be associated with increased lupus susceptibility, in association with disease severity [30], as well as with more advanced disease stages of colorectal cancer (CRC) [31]. Nevertheless, data on *IGF1R* gene variations in SS—a disease model of the crossroad between autoimmunity and malignancy—are absent.

In the current study, we aimed to investigate whether the *IGF1R* gene polymorphism rs2229765 is implicated in SS pathogenesis and explore potential functional pathogenetic implications.

## 2. Materials and Methods

### 2.1. Patients

In the present case–control study, DNA derived from peripheral blood of 277 primary SS patients [77 complicated with lymphoma (SS-lymphoma) and 200 without lymphoma (SS)] and 337 healthy individuals (HCs), extracted and stored in the Molecular and Applied Physiology Unit, Department of Physiology, National and Kapodistrian University of Athens, was implemented for genotype studies. Moreover, RNA was extracted from 56 labial MSG biopsies [42 derived from patients fulfilling ACR/EULAR SS classification criteria [32] and 14 from patients presenting with sicca complaints (SC)]. All biopsies were performed as part of the routine diagnostic evaluation of SS. All primary SS patients who fulfilled the 2016 SS classification criteria were followed up by CPM and HMM, while SS patients who were complicated by lymphoma were followed up by MV. All lymphomas were of mucosa-associated lymphoid tissue (MALT) type, and the diagnosis was based on World Health Organization classification criteria [33]. Patients younger than 18 years were excluded from the study. Clinical, serological, and histopathological characteristics were available for all patients, as described in a previous study approved by the Ethics Committee of the National and Kapodistrian University of Athens (protocol code: 1516031811; date of approval: 7 July 2016) [34]. Informed consent was obtained from all subjects ahead of study entry in accordance with the Declaration of Helsinki.

### 2.2. DNA Extraction

Genomic DNA was extracted from whole blood samples using the Nucleospin Blood QuickPure kit (Macherey-Nagel GmbH & Co, Düren, Germany), according to the manufacturer’s instructions. DNA concentration was measured by a Biospec-Nano spectrophotometer (Shimadzu, Kyoto, Japan).

### 2.3. Restriction Fragment Length Polymorphism–Polymerase Chain Reaction (RFLP–PCR)

DNA extracted from primary SS patients and HCs was genotyped for the IGF1R rs2229765 single nucleotide polymorphism variant [30] by RFLP-PCR. Briefly, a genomic DNA aliquot (about 30–50 ng/mL) was amplified by polymerase chain reaction (PCR) using the following primers: forward 5’-TCTTCTCCAGTGTACGTTCC-3’and reverse 5′-GGAACTTTCTCTTTACCACATG-3’. PCR amplification was performed in a Gene Amp PCR System 9700 (Applied Biosystems, Foster City, CA, USA) for 35 cycles (30 s at 94 °C, 30 s at 58 °C, and 30 s at 72 °C). The resulting PCR products of 255 bp were digested using 2.5 units of restriction endonuclease MnlI (New England Biolabs) per reaction for 55 s at 37 °C. Kits for PCR reactions were supplied by Kapa Biosystems (Kapa Ready Mix). PCR products and restriction fragments were visualized on a 3% agarose gel stained with ethidium bromide. The +3179G allele yielded four fragments: 132, 80, 23, and 20 bp; the +3179A allele yielded three fragments: 132, 100, and 20 bp.

### 2.4. cDNA Synthesis and Real-Time PCR

Total RNA obtained from MSG samples was reverse-transcribed using the Superscript III reverse transcriptase system from Invitrogen (Thermo Fisher, Waltham, MA, USA). Complementary DNA samples were diluted 1:10 with nuclease-free water (Qiagen, Germany) immediately after synthesis and stored at −20 °C. Quantitative real-time polymerase chain reaction (qRT-PCR) was used to quantify specific cDNAs using the Bio-Rad IQ5 thermocycler and Kapa Biosystems SYBR Green (Kapa Biosystems, Cape Town, South Africa). Specific primers to amplify only cDNA (exon-intron spanning) for each gene were designed using the Beacon Designer software. The sequences of each primer set for glyceraldehyde phosphate dehydrogenase (GAPDH), IGF1R, IGFIEa, IGFIEb, IGFIEc, IGFBP3, IGFBP6, caspase 1, caspase 4, caspase 5, NLRP3, apoptosis-associated speck-like (ASC), interleukin-1β (IL1β), IL18, and IL33, respectively, are presented in Table S1. As an internal control and normalization gene, we used the GAPDH gene. The reaction was carried out in a total volume of 20 μL per reaction and was constituted of 2 μL of template cDNA, 0.4 μM of each primer, 10 μL 2× KAPA SYBR Green Mix (Kapa Biosystems, South Africa), and ultra-pure water. A two-step amplification protocol was applied, starting with Step 1, with one cycle at 95 °C for 4 min, followed by Step 2, with 40 cycles at 95 °C for 5 s and 63 °C for 30 s. The specificity of the amplified products was determined by melting curve analysis.

The threshold cycles (Cts) generated by the qPCR system were used to calculate relative gene expression levels between different samples. Briefly, the Ct of the target gene (or gene of interest) was subtracted from the Ct of the reference gene for the two groups. Then, the relative expression of each sample was determined using the 2^−ΔΔCt^ method, as previously described [4]. All reactions were performed in duplicate. The MSG healthy RNA pool was the reference sample for MSG tissues.

### 2.5. Immunohistochemistry

Immunohistochemical detection of IGF1R was performed by a standard immunoperoxidase technique using the SignalStain^®^ Boost IHC detection reagent (HRP, rabbit; Cell Signaling, Danvers, MA, USA) in 10 formalin-fixed paraffin-embedded MSG tissue sections (5 μm) derived from 5 SC and 10 SS patients. Briefly, the paraffin sections were rehydrated in successive baths of xylene, 100%, 96%, 80%, 70% ethanol, and distilled water. The sections were washed with PBS (phosphate-buffered saline) 3 times. Antigen retrieval was performed by microwaving for 10 min in 0.01 M citrate buffer (pH 6.0). Incubation for 10 min at room temperature with Power Block Universal Blocking Reagent (Cell Signalling, Danvers, MA, USA) and 10 min with 3% H_2_O_2_ (BioGenex, San Ramon, CA, USA) was performed to block non-specific antibody binding and endogenous peroxidase activity, respectively. Incubation of serial sections with IGF1R Rabbit Polyclonal Antibody (Origene, Rockville, MD, USA) at 1:100 dilution and concentration-matched isotype control antibody (PharMingen, San Diego, CA, USA) was performed for 30 min at room temperature. Polymer–horseradish peroxidize (HRP) reagent (Cell Signaling, USA) was applied for 30 min at room temperature, and, after a washing step, substrate diaminobenzidine (DAB) solution (Cell Signalling, USA) was applied for 5–10 min. Biopsy sections were counterstained with hematoxylin for 2 min (Mayers hematoxylin solution, Sigma Aldrich Inc, Burlington, MA, USA), dehydrated in successive baths of water, 70%, 80%, 96%, 100% ethanol, and xylene and coverslip-mounted with two drops of aqueous mounting media (Chembiotin, Canada). Negative control staining was performed by replacing the primary antibody with PBS. Positive immunoreactivity appears as brown color. A human breast cancer section sample was used as positive control, available from our laboratory, according to the IGF1R Rabbit Polyclonal Antibody (Origene, USA) datasheet. Positive immunoreactivity appears as brown color. The intensity of immunoreactivity for IGF1R was determined as follows: 0 for no staining, 1 for weak intensity staining, 2 for intermediate intensity staining, and 3 for strong intensity staining, as previously suggested [35]. 

### 2.6. Statistics

Allele and genotype frequencies in patients and HCs were determined for the IGF1R rs2229765 variant by SHEsis and SNPStats software. Genotype frequencies in control subjects for each SNP were tested for departure from the Hardy–Weinberg equilibrium. Genotype frequencies for the rs2229765 variant were compared in patients and HCs using the χ^2^ test and ORs, and corresponding 95% CIs were estimated. Adjustment for the effects of age and gender was performed. Five genetic models (codominant, dominant, recessive, overdominant, and additive) were also determined.

We assessed two-group comparisons of continuous data using *t*-tests or the Mann-Whitney test when the data distribution was not normal. The SPSS v.26 and GraphPad Prism 5 software were used. We determined the correlation between gene expression data using a non-parametric Spearman’s test. A *p*-value of <0.05 was considered statistically significant.

## 3. Results

### 3.1. Clinical and Serological Characteristics of Study Participants

Clinical, hematological, serological, and histopathological characteristics of SS-non lymphoma (SS-nL) and SS-lymphoma (SS-L) patients included in the study are displayed in Table S2. The mean age ± standard deviation (SD) at disease onset was 52.5 ± 12.9 years in the SS group and 51.7 ± 13.8 in the SS-L group. The prevalence of females in the SS and SS-L cohorts was 93.2% and 89.6%, respectively. In the HC group, the age at study entry was 55.5 ± 9 years, and the prevalence of females was 93.8%.

### 3.2. Allele and Genotype Analysis in SS, SS-Lymphoma Patients, and HCs

To investigate the allele and genotype distribution of *IGF1R* gene polymorphism rs2229765 among SS and SS-L patients and HCs, allele and genotype analyses were performed. The A allele was found to be significantly more frequent in the whole SS population included in the study compared to HCs (44.6% vs. 38.7%, OR [95% CI]: 1.27 [1.01–1.60], *p* = 0.04). A marginally significant difference was found between SS-nL patients and HCs (44.5% vs. 38.7%, OR [95% CI]: 1.27 [0.99–1.60], *p* = 0.06). No significant differences were detected between the SS-L group compared to the HC or SS-nL group (Table 1).

Next, we compared the prevalence of the A/A genotype among HC, SS, and SS-L patients after adjustment for age and sex. The prevalence of the A/A genotype was found to be significantly higher in the whole SS population compared to HCs for both codominant and recessive models (22.4% vs. 10.7%, OR [95% CI]: 2.30 [1.17–4.52], *p* = 0.005 and 2.61 [1.42–4.80], *p* = 0.002, respectively), as well as between SS-nL patients and HCs (22.4% vs. 10.7% in the codominant model, with OR [95% CI]: 2.42 [1.15–5.12], *p* = 0.012, and the recessive model, with OR [95% CI]: 2.68 [1.37–5.26], *p*= 0.004). No significant differences in the prevalence of the A/A genotype were observed between SS-L patients and HCs (Table 2).

### 3.3. Clinical and Laboratory Associations of the IGF1R Polymorphism rs2229765 among SS Patients

Given the increased prevalence of the A/A genotype in SS patients compared to HCs, we next sought to explore whether the A/A genotype of the IGF1R rs2229765 variant is associated with distinct clinical, serological, or histopathological features. SS patients carrying the A/A genotype displayed a higher, though marginally significant, frequency of anti-Ro/SSA positivity compared to those harboring the A/G and G/G genotypes (86.7% vs. 75.2%, *p* = 0.06), lower monocyte absolute numbers (337 ± 156 vs. 413 ± 197, *p* = 0.016), lower hemoglobin (HGB) concentrations (12.0 ± 1.3 vs. 13.0 ± 4.1 g/dL, *p* = 0.013), and younger age at disease diagnosis (49.4 ± 13.6 vs. 53.0 ± 13.0, *p* = 0.035). No other significant associations between the A/A genotype of the IGF1R rs2229765 variation with clinical or laboratory features were detected (Table 3). After stratification of SS patients according to the presence or not of the A allele (A/G-A/A vs. GG), a significantly higher IGFIEa gene expression in MSG tissues (6.0 ± 2.5 vs. 3.0 ±3.3, *p* = 0.016) and B-cell activating factor (BAFF) serum protein levels (1774 ± 1529 vs. 868.5 ± 309.4 pg/mL, *p* = 0.03, measured in a previous study [36]) was found in A allele carriers compared to non-carriers (data not shown). No significant associations between A allele carriers and IGF1R gene expression at the MSG tissue level or peripheral blood were demonstrated (data not shown).

### 3.4. Allele and Genotype Analyses in SS, SS-Lymphoma Patients According to Anti-Ro/SSA Status and HCs

**Figure 1:**
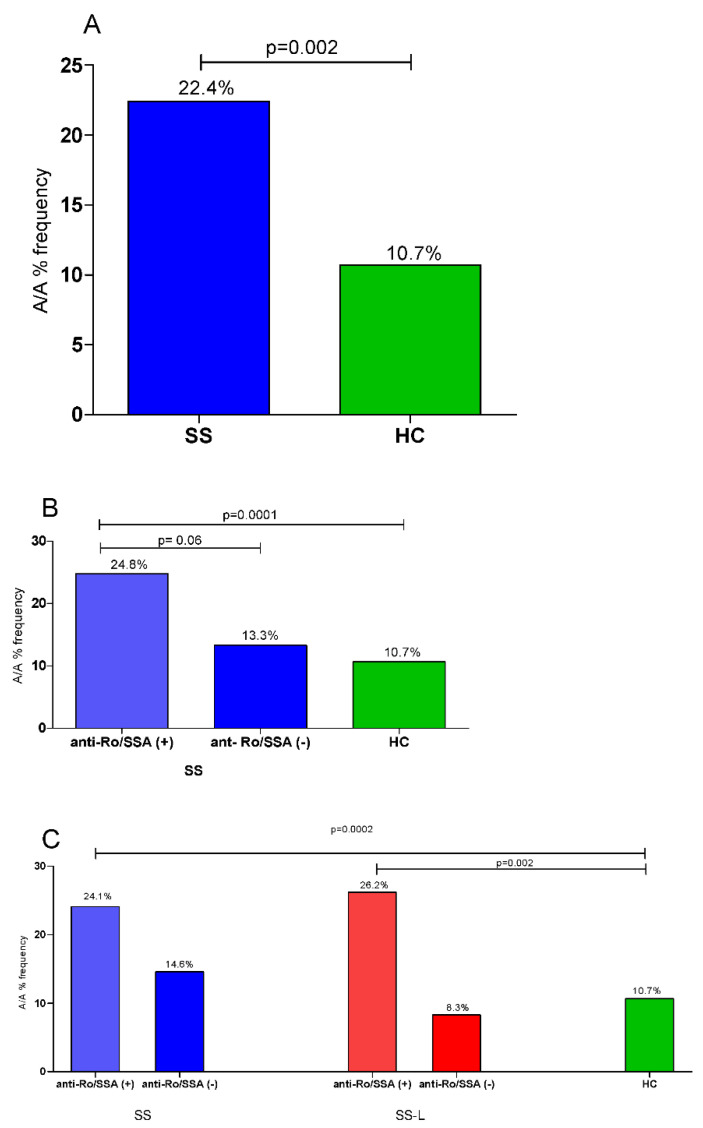
Frequency of the A/A genotype of the IGF1R rs2229765 variant in SS and SS-L patients compared to HCs according to anti-Ro/SSA status. (**A**) Significantly higher frequency of the A/A genotype in the whole SS population compared to HCs (22.4% vs. 10.7%, *p* = 0.002). (**B**) Significantly increased frequency of the A/A genotype in anti-Ro/SSA positive but not the anti-Ro/SSA negative SS population compared to HCs (24.8% vs. 10.7%, *p* = 0.0001, and 13.3% vs. 10.7%, *p* ˃ 0.05, respectively). (**C**) Both anti-Ro/SSA positive SS and SS-L patients displayed increased frequency of the A/A genotype compared to HCs (24.1% vs. 10.7%, *p* = 0.0002, and 26.2% vs. 10.7%, *p* = 0.002, respectively). SS: Sjogren’s syndrome; SS-L: Sjogren’s syndrome complicated by lymphoma; HC: healthy control group.

Given the identified difference in the frequency of anti-Ro/SSA between patients carrying the A/A genotype vs. those harboring the A/G and G/G genotypes, we next stratified SS patients according to anti-Ro/SSA and lymphoma status. As shown in Figure 1, the difference between the whole SS population vs. HC (22.4% vs. 10.7%, *p* = 0.002, panel A) is mainly attributed to differences detected between the anti-Ro/SSA positive [24.8% vs. 10.7%, *p* = 0.0001, OR 95% CI: 2.8 (1.7–4.4)], rather than the anti-Ro/SSA negative group compared to the HC group (13.3 vs. 10.7, *p* > 0.05, panel B). Of interest, both anti-Ro/SSA positive SS and SS-L patients displayed increased frequency of the A/A genotype compared to HCs (24.1% vs. 10.7%, *p* = 0.0002, and 26.2% vs. 10.7%, *p* = 0.002, respectively).

### 3.5. Gene and Protein Expression of IGF1/IGF1R Axis Components in MSG Tissues

Given that the IGF1R gene variant has been shown to increase susceptibility for seropositive SS-nL and SS-L, we next sought to explore whether IGF1R expression is altered in peripheral blood and MSG tissues from these patients.

Although non-significant differences were detected between SS patients and SCs in peripheral blood (Figure S1A), IGF1R expression in peripheral blood was negatively correlated with the erythrocyte sedimentation rate (ESR) and IgG serum levels (r = −0.390, *p* = 0.001, and −0.578, *p* = 0.030, respectively) and positively correlated with urine-specific gravity values (r = 0.697, *p* = 0.001). No other significant associations were detected in peripheral blood IGF1R expression and clinical or serological disease-related features (data not shown).

**Figure 2:**
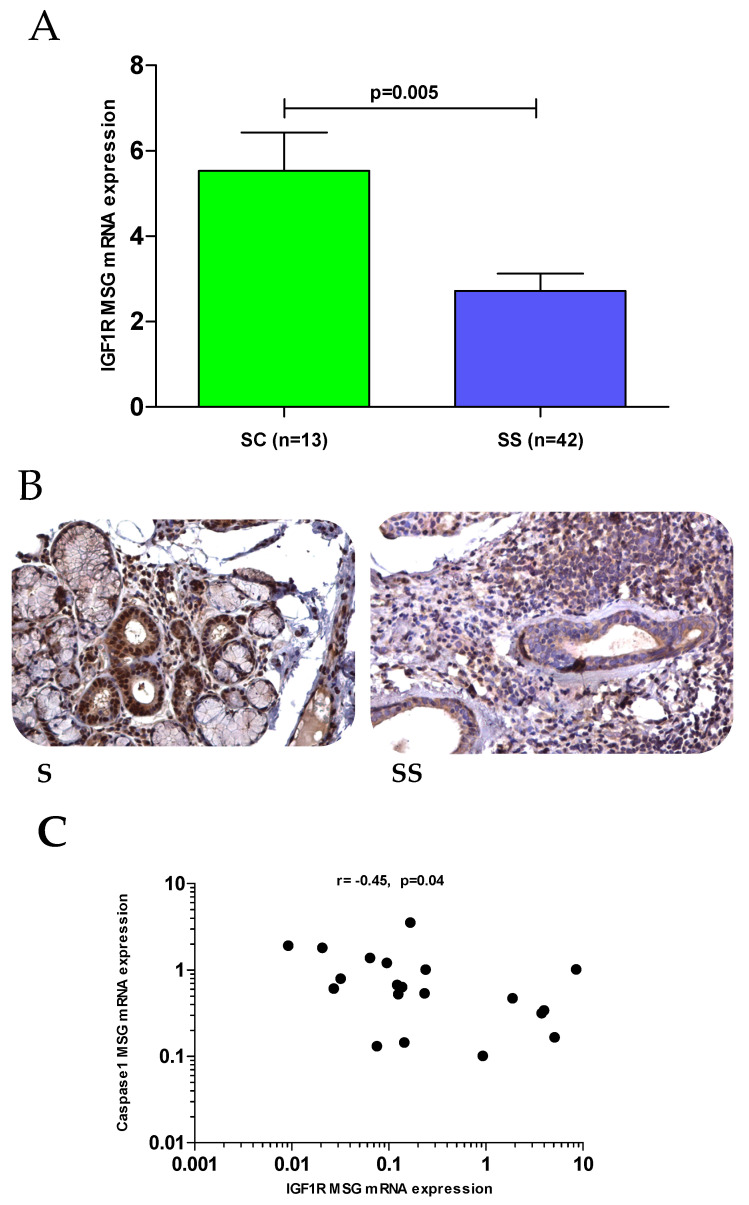
IGF1R mRNA (measured by real-time PCR) and immunohistochemical expression in SC vs. SS patients. (**A**) IGF1R transcript levels were significantly lower in MSG tissues derived from SS patients compared to SCs (2.7 ± 2.7 vs. 5.5 ± 3.2, *p* = 0.005). (**B**) Representative images depicting reduced IGF1R immunohistochemical expression in salivary ducts and acini of MSG tissue sections from a patient with SS compared to SCs (40×). (**C**) Negative correlation between IGF1R transcripts caspase 1 mRNA expression in MSGs derived from SS patients (r = −0.45, *p* = 0.04). SS: Sjogren’s syndrome; MSG: minor salivary gland; IGF1R: insulin growth factor receptor 1; r: Spearman’s correlation.

Compared to MSG tissues derived from SCs, SS MSG biopsies displayed significantly reduced mRNA (2.7 ±2.7 vs. 5.5 ± 3.2, *p* = 0.005) (Figure 2A) and immunohistochemical protein expression mainly in salivary ducts and acini (Figure 2B). No significant differences in the other components of the IGF1/IGF1R axis, including IGFIEa (0.9 ± 1 vs. 1 ± 1.4, *p* = 0.55), IGFIEb (3 ± 3.3 vs. 5.6 ± 9.5, *p* = 0.67), IGFIEc (1.6 ± 4.3 vs. 2.1 ± 3.8, *p* = 0.87), IGFBP3 (1.4 ± 2.3 vs. 0.9 ± 1.6, *p* = 0.40), and IGFBP6 (2.3 ± 2.1 vs. 1.6 ± 0.7, *p* = 0.80) mRNA expression, were detected in MSG biopsies derived from SS patients compared to SCs (Figure S1).

### 3.6. Dampened IGF1R Expression and Apoptotic Pathways

**Figure 3:**
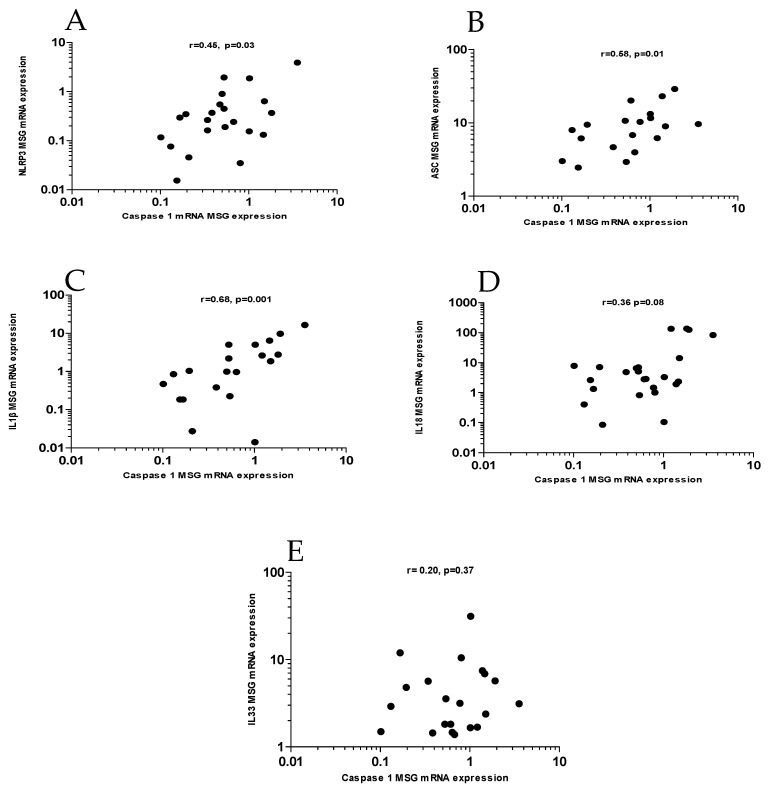
IGF1R transcripts, inflammatory apoptotic molecules, and inflammasome components in salivary gland tissues from patients with SS. Significant correlations between caspase 1 gene expression with NLRP3 (r = 0.45, *p* = 0.03) (**A**), ASC (r = 0.58, *p* = 0.01) (**B**), IL1β (r = 0.68, *p* = 0.001) (**C**). No associations between IL18 or IL33 with caspase 1 mRNA expression were detected (**D**,**E**). SS: Sjogren’s syndrome; MSG: minor salivary gland; NLRP3: NOD-like receptor pyrin domain containing 3; ASC: apoptosis-associated speck-like; r: Spearman’s correlation.

Given the well-established effects of the IGF1/IGF1R axis in cell growth and survival [37,38,39], we hypothesized that dampened IGF1R expression in SS MSG tissues could be related to apoptotic events in SS salivary gland epithelial cells, as previously described [14]. Towards this end, gene expression of several apoptotic molecules was determined, including caspases 1, 4, and 5, as well as NLRP3- inflammasome components (NLRP3, ASC, IL1β, IL18, IL33). Of interest, IGF1R mRNA expression was only found to be significantly inversely correlated with caspase 1 mRNA expression (r = −0.45, *p* = 0.04) in SS-derived MSGs (Figure 2C). In turn, as displayed in Figure 3, caspase 1 mRNA expression in MSG tissues was significantly associated with several components of the inflammasome axis, including NLRP3 (r = 0.45, *p* = 0.03, panel A), ASC (r = 0.58, *p* = 0.01, panel B), and IL1β (r = 0.68, *p* = 0.001, panel C). No significant associations were detected between caspase 1 with IL18 (r = 0.36, *p* = 0.08, panel D) or IL33 MSG tissue transcripts (r = 0.20, *p* = 0.37, panel E). As shown in Figure S2, and in accord with previous reports [18,19], inflammasome components were upregulated in SS patients compared to SCs (Figure S2).

## 4. Discussion

In the present study, we aimed to explore a potential contributory role of the IGF1/IGF1R pathway in the pathogenesis of SS. In this context, we report a novel association of the A/A genotype of the IGF1R gene variant rs2229765 with seropositive SS irrespective of lymphoma status. Carriers of the A allele were shown to be younger at disease diagnosis, displaying lower monocyte absolute numbers and Hgb levels as well as increased IGFIEa MSG mRNA expression and serum BAFF protein levels compared to non-carriers. Moreover, in comparison to SCs, SS patients displayed reduced mRNA and protein (ductal/acinar) expression in MSG tissues, which was negatively correlated with caspase 1 transcripts. The latter was shown to positively correlate with inflammasome components and members of the IL1 pathway at the salivary gland tissue level. No associations between A allele carriers with IGF1R expression were detected in peripheral blood or salivary gland tissues. Of note, IGF1R expression in peripheral blood was negatively correlated with ESR and IgG serum levels and positively correlated with urine-specific gravity values.

Together, these data could imply that defective growth signals to SGECs related to IGF1R downregulation could lead to increased pyroptosis, the release of intracellular autoantigens such as Ro/SSA, the initiation of a local inflammatory immune response in salivary gland tissue, and the production of anti-Ro/SSA antibodies. Indeed, apoptosis of SGECs is considered an essential pathogenetic mechanism for SS development through the accumulation of apoptotic debris and autoantigen discharge, which can activate the immune response [15,40]. In accord with these findings, it has been recently reported that increased plasma levels of pro-apoptotic molecules, including Bcl2, Fas, and FasL, are associated with more severe inflammation status in SS patients than controls [41]. Moreover, overexpression of lysosome-associated membrane glycoprotein 3 (LAMP3)—a lysosomal protein playing a central role in antigen presentation and cell survival—in SS-derived MSGs has been associated with increased SGEC apoptosis and higher serum autoantibodies in SS patients [42]. Additionally, SGEC-derived exosomes containing autoantigens such as Ro/SSA could represent a potential source of immunogenic autoantigens [40,43].

Reduced immunohistochemical detection of IGF1R in both an experimental autoimmune sialadenitis animal model [26] and salivary gland biopsies derived from SS patients [27], as well as decreased IGF1R gene expression in peripheral blood monocytes [29], has been previously described. Given the anti-apoptotic role of IGF1, the downregulation of IGF1R and subsequential interruption of survival signals could contribute to increased apoptosis of SGECs, leading to the organization of the local inflammatory response and ensuing gland dysfunction. Moreover, in line with a growing body of data supporting a key role of inflammasome activation in the development of SS and SS-related lymphoma [18,19,44], we have identified the upregulation of many inflammasome components in salivary gland tissues derived from SS patients compared to controls. The negative correlation between IGF1R and caspase 1 provides a potential link between IGF1R downregulation, caspase 1 activation, and, subsequently, the activation of the IL1 pathways. Recent studies suggest that the NLRP3 inflammasome is a major activating factor of caspase 1, leading to IL1β and IL18 oversecretion and, finally, to the activation of the forming-pore enzyme gasdermin D, resulting in cell membrane rupture and driving inflammatory cell death (named pyroptosis) [45]. Moreover, caspase 4 and 5—shown to be upregulated in SS MSG samples compared to SCs—are considered central executors of non-canonical caspase-independent pyroptosis, also leading to IL1β and IL18 release [46].

The reason for the observed IGF1R downregulation is not clear. Earlier, in vitro studies using a human salivary adenocarcinoma cell line have shown that proinflammatory cytokines TNF-α and IFN-γ, which are highly expressed in SS salivary glands, are capable of inhibiting cell growth [47,48] as well as causing the downregulation of IGF1R, suppressing the promoter of the gene [49]. More recent studies have shown that miRNAs—also linked to autoimmunity development—are important regulators of IGF1R expression [50]; however, their role should be further investigated. To explore whether the rs2229765 variant is associated with differential IGF1R gene expression in MSG tissues or peripheral blood, we stratified the available samples according to the rs2229765 genotype. No differences in IGF1R mRNA expression were detected among SS patients in all comparison groups in MSGs or peripheral blood. The G/A variant of rs2229765, located in the exon area, is a synonymous polymorphism leading to an azotate base change from guanine to adenosine, resulting in the amino acid change from Gly (glycine) to Glu (glutamic acid). at position 1043 of the polypeptide chain. Thus, it is less probable that this change causes a functional alteration in IGF1R [51], while it has been shown that it is able to regulate the alternative splicing of IGF1R mRNA [52]. Therefore, further studies are needed to determine the functional effects of IGF1R gene variant rs2229765.

Our genetic findings are somewhat in line with a previous study conducted on a Bulgarian SLE cohort, reporting that the SLE carriers of the A/A genotype and A allele displayed an increased (although statistically non-significant) risk for SLE susceptibility compared to their G/G-G/A counterparts [30]. Regarding the role of IGF1R gene variant rs2229765 in cancer development, the current understanding remains controversial. Previous studies have shown that the A allele was associated with advanced colorectal cancer [31] and increased breast cancer risk [53]. At the same time, no association was found with non-small cell lung cancer [54] or breast cancer survival [51]. On the contrary, the A allele seems to display a protective role in papillary thyroid carcinoma [55] and melanoma development [56]. In our cohort, the presence of the A/A genotype has been shown to confer increased susceptibility for both anti-Ro/SSA positive SS and SS-related lymphoma, but not for their anti-Ro/SSA negative counterparts compared to healthy controls. Furthermore, we have shown that A allele carriers are younger at disease diagnosis, display lower monocyte absolute numbers and Hgb, as well as increased IGFIEa mRNA expression in MSG tissues and serum BAFF protein levels compared to non-carriers. Taken into consideration that serum anti-Ro/SSA [57], earlier disease onset [58], lower HGB values [59], and heightened serum BAFF levels [4] have all been previously associated with lymphoma development among SS patients, it seems that alterations of the IGF1/IGF1R axis could be significant contributors of severe inflammation and malignant transformation in the setting of SS.

## 5. Conclusions

In the present study, the rs2229765 IGF1R variant has been shown to increase susceptibility for seropositive primary SS and primary SS-related lymphoma. Decreased trophic signals in salivary gland epithelial cells due to dampened IGF1R mRNA and protein expression in salivary gland tissues could be related to increased apoptosis and, subsequently, to the activation of inflammasome pathways in the setting of primary SS and SS-related lymphoma.

## Figures and Tables

**Table 1:** Distribution of the allelic frequencies of the IGF1R rs2229765 variant in SS populations versus HCs.

SNP	Allele	HC (*n* = 336) (%)	SS (*n* = 277) (%)	SS-nL (*n* = 200) (%)	SS-L (*n* = 77) (%)	* OR [95% CI] *p*-Value	** OR [95% CI] *p*-Value	*** OR [95% CI] *p*-Value
**rs2229765**	G	61.3%	55.4%	55.5%	55.2%	1.27 [1.01–1.60] 0.04	1.27 [0.99–1.60] 0.06	1.28 [0.90–1.80] 0.16
	A	38.7%	44.6%	44.5%	44.8%			

* SS vs. HC, ** SS-nL vs. HC, *** SS-L vs. HC. SS: Sjögren’s syndrome; SS-nL: SS non-lymphoma; SS-L: SS-lymphoma; OR: odds ratio; 95% CI: 95% confidence interval; HC: healthy controls, SNP: single nucleotide polymorphism.

**Table 2:** Genotypic frequencies of the IGF1R rs2229765 variant in SS patients with or no lymphoma versus HCs after adjustment for age and sex.

SNP	Model	Genotype	HC (*n* = 337)	SS (*n* = 277)	SS-nL (*n* = 200)	SS-L (*n* = 77)	* OR [95% CI]	** OR [95% CI]	*** OR [95% CI]	*p*-Value	*p*-Value	*p*-Value
**rs2229765**	Codominant	G/G	33.2 %	33.2%	33.2%	33.8%	1.00	1.00	1.00	0.005	0.012	0.27
		G/A	56.1%	44.4%	44.4%	42.9%	0.81 (0.49–1.32)	0.80 (0.48–1.48)	0.92 (0.44–1.95)			
		A/A	10.7%	22.4%	22.4%	23.4%	2.30 (1.17–4.52)	2.42 (1.15–5.12)	2.11 (0.73–6.08			
	Dominant	G/G	33.2%	33.2%	33%	33.8%	1.00	1.00	1.00	0.83	0.7	0.8
		G/A-A/A	66.8%	66.8%	67%	66.2%	1.05 (0.66–1.67)	1.11 (0.66–1.89)	1.10 (0.54–2.23)			
	Recessive	G/G-G/A	89.3%	77.6%	78%	76.6%	1.00	1.00	1.00	0.002	0.004	0.11
		A/A	10.7%	22.4%	22.4%	23.4%	2.61 (1.42–4.80)	2.68 (1.37–5.26)	2.21 (0.84–5.79)			
	Overdominant	G/G-A/A	43.9 %	55.6%	55%	57.1%	1.00	1.00	1.00	0.033	0.07	0.4
		G/A	56.1 %	44.4%	45%	42.9%	0.62 (0.40–0.96)	0.63 (0.38–1.04)	0.75 (0.38–1.47			
	Log-additive						1.35 (0.98–1.86)	1.41 (0.98–2.02)	1.31 (0.78–2.19)	0.07	0.06	0.31

* SS patients vs. HC, ** SS-nL patients vs. HC, *** SS- L patients vs. HC, SS: Sjögren’s syndrome; SS non-lymphoma; SS-L: SS-lymphoma; OR: odds ratio; 95% CI: 95% confidence interval; HC: healthy control group, SNP: single nucleotide polymorphism.

**Table 3:** Demographic, clinical, histopathological, and serological features of SS patients carrying the G/G-A/A genotype and SS patients carrying the A/A genotype.

Clinical/Serological Characteristics of the SS Cohort			
	Within G/G-G/A Genotype	Within A/A Genotype	*p*-Values
**Demographics**			
Mean age (years) (± SD)	58.4 ± 12.6	58.6 ± 15.0	ns
Gender/Female, *n* (%)	90.9	96.8	ns
Age at SS diagnosis (mean ± SD)	53.0 ± 13.0	49.4 ± 13.6	0.035
**Clinical features**			
Dry mouth, *n* (%)	93.9	95.2	ns
Dry eyes, *n* (%)	88.2	90.5	ns
Salivary gland enlargement, *n* (%)	30.8	43.5	0.06
Rose-Bengal stain, *n* (%)	57.7	54.8	ns
Abnormal Schirmer’s test	81.3	86.4	ns
Arthralgias-Myalgias, *n* (%)	64.9	68.3	ns
Raynaud’s phenomenon, *n* (%)	27.1	31.7	ns
Palpable Purpura, *n* (%)	15.2	15.9	ns
**Serological Features**			
Anti-Ro/SSA, *n* (%)	75.2	86.7	0.06
Anti-La/SSB, *n* (%)	39.5	41.7	ns
Rheumatoid Factor positivity, (> 20 IU/mL) (%)	56.4	68.5	ns
WBC (mean ± SD/mm^3^)	5633 ± 1967	5543 ± 2281	ns
Neutrophils (mean ± SD/mm^3^)	3363 ±1711	3366 ± 1696	ns
Monocytes (mean ± SD/mm^3^)	413 ± 197	337 ± 156	0.016
Lymphocytes (mean ± SD/mm^3^)	1631 ± 687	1696 ± 789	ns
HGB g/dL (mean ± SD)	13 ± 4.08	12 ± 1.28	0.013
Erythrocyte sedimentation rate SR mm/hour (mean ± SD)	31.63 ± 24,75	35.74 ± 29.32	ns
IgG mg/dL (mean ± SD)	1687.07 ± 785.86	1884.66 ± 1016.98	ns
IgM mg/dL (mean ± SD)	159.24 ± 125.24	236.49 ± 281.73	ns
IgA mg/dL (mean ± SD)	278.49 ± 118.73	337.25 ± 165.56	ns
LDH IU/L (mean ± SD)	237.91 ± 93.96	252.45 ± 111.15	ns
C3 mg/dL (mean ± SD)	112.29 ± 66.54	103.43 ± 25.72	ns
C4 mg/dL (mean ± SD)	19.16 ± 9.44	16.81 ± 7.11	ns
**Histopathological features**			
Focus score number of lymphocytic infiltrates/4 mm^2^ (mean ± SD)	2.41 ± 2.49	2.06 ± 1.47	ns

WBC: white blood cells; HGB: hemoglobin; LDH: lactate dehydrogenase; ns: not significant.

## Data Availability

The data presented in this study are available on request from the corresponding author.

## Supplementary Materials

The following are available online at https://www.mdpi.com/article/10.3390/jcm10173960/s1: Supplementary Table S1: Primer sequences used for quantitative Real-Time PCR reaction., Supplamentary Table S2: Demographic, clinical and laboratory features of SS patients according to lymphoma status., Supplementary Figure S1: IGF1/IGF1R axis components mRNA expression (measured by Real-Time PCR), in MSG tissues derived from SS patients and SC, Supplementary Figure S2: Inflammatory apoptosis components mRNA expression (measured by Real-Time PCR) in MSG tissues derived from SS patients compared to SC.

## References

[1] Mavragani C.P., Moutsopoulos H.M. (2014). Sjögren Syndrome Review. Can. Med. Assoc. J..

[2] Mariette X., Criswell L.A. (2018). Primary Sjögren’s Syndrome. N. Engl. J. Med..

[3] Qin B., Wang J., Yang Z., Yang M., Ma N., Huang F., Zhong R. (2015). Epidemiology of primary Sjögren’s syndrome: A systematic review and meta-analysis. Ann. Rheum. Dis..

[4] Nezos A., Gravani F., Tassidou A., Kapsogeorgou E.K., Voulgarelis M., Koutsilieris M., Crow M.K., Mavragani C.P. (2015). Type I and II interferon signatures in Sjogren’s syndrome pathogenesis: Contributions in distinct clinical phenotypes and Sjogren’s related lymphomagenesis. J. Autoimmun..

[5] Bodewes I.L.A., Versnel M.A. (2018). Interferon activation in primary Sjögren’s syndrome: Recent insights and future perspective as novel treatment target. Expert Rev. Clin. Immunol..

[6] Huijser E., Versnel M. (2021). Making Sense of Intracellular Nucleic Acid Sensing in Type I Interferon Activation in Sjögren’s Syndrome. J. Clin. Med..

[7] Cinoku I.I., Verrou K.-M., Piperi E., Voulgarelis M., Moutsopoulos H.M., Mavragani C.P. (2021). Interferon (IFN)-stimulated gene 15: A novel biomarker for lymphoma development in Sjögren’s syndrome. J. Autoimmun..

[8] Zintzaras E., Voulgarelis M., Moutsopoulos H.M. (2005). The Risk of Lymphoma Development in Autoimmune Diseases: A Meta-Analysis. Arch. Intern. Med..

[9] Stergiou I.E., Poulaki A., Voulgarelis M. (2020). Pathogenetic Mechanisms Implicated in Sjögren’s Syndrome Lymphomagenesis: A Review of the Literature. J. Clin. Med..

[10] Skarlis C., Argyriou E., Mavragani C.P. (2020). Lymphoma in Sjögren’s Syndrome: Predictors and Therapeutic Options. Curr. Treat. Options Rheumatol..

[11] Verstappen G.M., Pringle S., Bootsma H., Kroese F.G.M. (2021). Epithelial–immune cell interplay in primary Sjögren syndrome salivary gland pathogenesis. Nat. Rev. Rheumatol..

[12] Psianou K., Panagoulias I., Papanastasiou A.D., De Lastic A.-L., Rodi M., Spantidea P.I., Degn S.E., Georgiou P., Mouzaki A. (2018). Clinical and immunological parameters of Sjögren’s syndrome. Autoimmun. Rev..

[13] Mavragani C.P., Fragoulis G.E., Moutsopoulos H.M. (2012). Endocrine alterations in primary Sjogren’s syndrome: An overview. J. Autoimmun..

[14] Sisto M., Lisi S., Lofrumento D.D., D’Amore M., Scagliusi P., Mitolo V. (2007). Autoantibodies from Sjogren’s Syndrome Trigger Apoptosis in Salivary Gland Cell Line. Ann. N. Y. Acad. Sci..

[15] Manganelli P., Fietta P. (2003). Apoptosis and Sjögren syndrome. Semin. Arthritis Rheum..

[16] Polihronis M., Tapinos N.I., Theocharis S.E., Economou A., Kittas C., Moutsopoulos H.M. (1998). Modes of epithelial cell death and repair in Sjögren’s syndrome (SS). Clin. Exp. Immunol..

[17] Manoussakis M.N., Boiu S., Korkolopoulou P., Kapsogeorgou E.K., Kavantzas N., Ziakas P., Patsouris E., Moutsopoulos H.M. (2007). Rates of infiltration by macrophages and dendritic cells and expression of interleukin-18 and interleukin-12 in the chronic inflammatory lesions of Sjögren’s syndrome: Correlation with certain features of immune hyperactivity and factors associated with high risk of lymphoma development. Arthritis Rheum..

[18] Vakrakou A.G., Boiu S., Ziakas P., Xingi E., Boleti H., Manoussakis M.N. (2018). Systemic activation of NLRP3 inflammasome in patients with severe primary Sjögren’s syndrome fueled by inflammagenic DNA accumulations. J. Autoimmun..

[19] Baldini C., Santini E., Rossi C., Donati V., Solini A. (2017). The P2X7 receptor-NLRP3 inflammasome complex predicts the development of non-Hodgkin’s lymphoma in Sjogren’s syndrome: A prospective, observational, single-centre study. J. Intern. Med..

[20] Blokland S.L.M., Flessa C.-M., van Roon J.A.G., Mavragani C.P. (2019). Emerging roles for chemokines and cytokines as orchestrators of immunopathology in Sjögren’s syndrome. Rheumatology.

[21] Adams T.E., Epa V.C., Garrett T.P.J., Ward C.W. (2000). Structure and function of the type 1 insulin-like growth factor receptor. Cell. Mol. Life Sci..

[22] Hjortebjerg R., Frystyk J. (2013). Determination of IGFs and their binding proteins. Best Pract. Res. Clin. Endocrinol. Metab..

[23] Hakuno F., Takahashi S.-I. (2018). 40 YEARS OF IGF1: IGF1 receptor signaling pathways. J. Mol. Endocrinol..

[24] Mitsui R., Fujita-Yoshigaki J., Narita T., Matsuki-Fukushima M., Satoh K., Qi B., Guo M.-Y., Katsumata-Kato O., Sugiya H. (2010). Maintenance of paracellular barrier function by insulin-like growth factor-I in submandibular gland cells. Arch. Oral Biol..

[25] Skarlis C., Nezos A., Mavragani C.P., Koutsilieris M. (2019). The role of insulin growth factors in autoimmune diseases. Ann. Res. Hosp..

[26] Mustafa W., Mustafa A., Elbakri N., Link H., Adem A. (2001). Reduced Levels of Insulin-Like Growth Factor-1 Receptor (IGF-1R) Suppress Cellular Signaling in Experimental Autoimmune Sialadenitis (EAS). J. Recept. Signal Transduct. Res..

[27] Katz J., Stavropoulos F., Cohen D., Robledo J., Stewart C., Heft M. (2003). IGF-1 and insulin receptor expression in the minor salivary gland tissues of Sjögren’s syndrome and mucoceles—Immunohistochemical study. Oral Dis..

[28] Markopoulos A.K., Poulopoulos A.K., Kayavis I., Papanayotou P. (2000). Immunohistochemical detection of insulin-like growth factor-I in the labial salivary glands of patients with Sjögren’s syndrome. Oral Dis..

[29] Emamian E.S., Leon J.M., Lessard C.J., Grandits M., Baechler E.C., Gaffney P., Segal B., Rhodus N.L., Moser K.L. (2009). Peripheral blood gene expression profiling in Sjögren’s syndrome. Genes Immun..

[30] Stanilova S., Ivanova M., Karakolev I., Stoilov R., Rashkov R., Manolova I., Stanilova S. (2013). Association of +3179G/A insulin-like growth factor-1 receptor polymorphism and insulin-like growth factor-1 serum level with systemic lupus erythematosus. Lupus.

[31] Stanilov N.S., Karakolev I.A., Deliysky T.S., Jovchev J.P., Stanilova S. (2014). Association of insulin-like growth factor-I receptor polymorphism with colorectal cancer development. Mol. Biol. Rep..

[32] Shiboski C.H., Shiboski S.C., Seror R., A Criswell L., Labetoulle M., Lietman T.M., Rasmussen A., Scofield H., Vitali C., Bowman S.J. (2016). 2016 American College of Rheumatology/European League Against Rheumatism classification criteria for primary Sjögren’s syndrome: A Consensus and Data-Driven Methodology Involving Three International Patient Cohorts. Ann. Rheum. Dis..

[33] Quintanilla-Martinez L. (2017). The 2016 updated WHO classification of lymphoid neoplasias. Hematol. Oncol..

[34] Argyriou E., Nezos A., Roussos P., Venetsanopoulou A., Voulgarelis M., Boki K., Tzioufas A., Moutsopoulos H., Mavragani C. (2021). Leukocyte Immunoglobulin-Like Receptor A3 (LILRA3): A Novel Marker for Lymphoma Development among Patients with Young Onset Sjogren’s Syndrome. J. Clin. Med..

[35] Meyerholz D.K., Beck A.P. (2018). Principles and approaches for reproducible scoring of tissue stains in research. Lab. Investig..

[36] Papageorgiou A., Mavragani C.P.C.P., Nezos A., Zintzaras E., Quartuccio L., De Vita S., Koutsilieris M., Tzioufas A.G.A.G., Moutsopoulos H.M., Voulgarelis M. (2015). A BAFF Receptor His159Tyr Mutation in Sjögren’s Syndrome-Related Lymphoproliferation. Arthritis Rheumatol..

[37] Denley A., Cosgrove L.J., Booker G.W., Wallace J.C., Forbes B.E. (2005). Molecular interactions of the IGF system. Cytokine Growth Factor Rev..

[38] Kooijman R. (2006). Regulation of apoptosis by insulin-like growth factor (IGF)-I. Cytokine Growth Factor Rev..

[39] Michalopoulou F., Petraki C., Philippou A., Analitis A., Msaouel P., Koutsilieris M. (2020). Expression of IGF-IEc Isoform in Renal Cell Carcinoma Tissues. Anticancer. Res..

[40] Mitsias D.I., Kapsogeorgou E.K., Moutsopoulos H.M. (2006). The role of epithelial cells in the initiation and perpetuation of autoimmune lesions: Lessons from Sjögren’s syndrome (autoimmune epithelitis). Lupus.

[41] Benchabane S., Slimani-Kaddouri A., Acheli D., Bendimerad-Iratene T., Mesbah R., Touil-Boukoffa C. (2021). Association between increased Bcl-2, Fas and FasL levels and inflammation extent in labial salivary glands during primary Sjögren’s syndrome. Endocr. Metab. Immune Disord. Drug Targets.

[42] Tanaka T., Warner B.M., Odani T., Ji Y., Mo Y.-Q., Nakamura H., Jang S.-I., Yin H., Michael D.G., Hirata N. (2020). LAMP3 induces apoptosis and autoantigen release in Sjögren’s syndrome patients. Sci. Rep..

[43] Kapsogeorgou E.K., Abu-Helu R.F., Moutsopoulos H.M., Manoussakis M.N. (2005). Salivary gland epithelial cell exosomes: A source of autoantigenic ribonucleoproteins. Arthritis Rheum..

[44] Kim S.-K., Choe J.-Y., Lee G.H. (2017). Enhanced expression of NLRP3 inflammasome-related inflammation in peripheral blood mononuclear cells in Sjögren’s syndrome. Clin. Chim. Acta.

[45] Lamkanfi M., Dixit V.M. (2010). Manipulation of Host Cell Death Pathways during Microbial Infections. Cell Host Microbe.

[46] Van Opdenbosch N., Lamkanfi M. (2019). Caspases in Cell Death, Inflammation, and Disease. Immunity.

[47] Katz J., Weiss H., Goldman B., Kanety H., Stannard B., Leroith D., Shemer J. (1995). Cytokines and growth factors modulate cell growth and insulin-like growth factor binding protein secretion by the human salivary cell line (HSG). J. Cell. Physiol..

[48] Katz J., Nasatzky E., Werner H., Roith D., Shemer J. (1999). Tumor necrosis factor α and interferon γ–induced cell growth arrest is mediated via insulin-like growth factor binding protein-3. Growth Horm. IGF Res..

[49] Shalita-Chesner M., Katz J., Shemer J., Werner H. (2001). Regulation of insulin-like growth factor-I receptor gene expression by tumor necrosis factor-α and interferon-γ. Mol. Cell. Endocrinol..

[50] Jung H.J., Suh Y. (2015). Regulation of IGF-1 signaling by microRNAs. Front. Genet..

[51] Deming S.L., Ren Z., Wen W., Shu X.O., Cai Q., Gao Y.-T., Zheng W. (2007). Genetic variation in IGF1, IGF-1R, IGFALS, and IGFBP3 in breast cancer survival among Chinese women: A report from the Shanghai Breast Cancer Study. Breast Cancer Res. Treat..

[52] De Alencar S.A., Lopes J.C.D. (2010). A Comprehensive In Silico Analysis of the Functional and Structural Impact of SNPs in theIGF1RGene. J. Biomed. Biotechnol..

[53] Kang H.-S., Ahn S.H., Mishra S.K., Hong K.-M., Lee E.S., Shin K.H., Ro J., Lee K.S., Kim M.K. (2014). Association of Polymorphisms and Haplotypes in the Insulin-Like Growth Factor 1 Receptor (IGF1R) Gene with the Risk of Breast Cancer in Korean Women. PLoS ONE.

[54] Gately K., Forde L., Gray S., Morris D., Corvin A., Tewari P., O’Byrne K. (2015). Mutational analysis of the insulin-like growth factor 1 receptor tyrosine kinase domain in non-small cell lung cancer patients. Mol. Clin. Oncol..

[55] Cho S.H., Kim S.K., Kwon E., Park H.J., Kwon K.H., Chung J.-H. (2012). Polymorphism ofIGF1RIs Associated with Papillary Thyroid Carcinoma in a Korean Population. J. Interf. Cytokine Res..

[56] Yuan T.-A., Yourk V., Farhat A., Guo K.L., Garcia A., Meyskens F.L., Liu-Smith F. (2020). A Possible Link of Genetic Variations in ER/IGF1R Pathway and Risk of Melanoma. Int. J. Mol. Sci..

[57] Fragkioudaki S., Mavragani C.P., Moutsopoulos H.M. (2016). Predicting the risk for lymphoma development in Sjogren syndrome: An Easy Tool for Clinical Use. Medicine.

[58] Goules A.V., Argyropoulou O.D., Pezoulas V.C., Chatzis L., Critselis E., Gandolfo S., Ferro F., Binutti M., Donati V., Callegher S.Z. (2020). Primary Sjögren’s Syndrome of Early and Late Onset: Distinct Clinical Phenotypes and Lymphoma Development. Front. Immunol..

[59] Flores-Chávez A., Kostov B., Solans R., Fraile G., Maure B., Feijoo-Massó C., Rascón F.-J., Pérez-Alvarez R., Zamora-Pasadas M., García-Pérez A. (2018). Severe, life-threatening phenotype of primary Sjögren’s syndrome: Clinical characterisation and outcomes in 1580 patients (GEAS-SS Registry). Clin. Exp. Rheumatol..

