# Supplementary material for: +3179G/A Insulin-Like Growth Factor-1 Receptor Polymorphism: A Novel Susceptibility Contributor in Anti-Ro/SSA Positive Patients with Sjögren’s Syndrome: Potential Clinical and Pathogenetic Implications
Source: J Clin Med. 2021 Aug 31;10(17):3960. doi: 10.3390/jcm10173960 (PMC8432056; doi:10.3390/jcm10173960)

Supplementary Material

Supplementary Table S1. Primer sequences used for quantitative Real-Time PCR reaction.

Primer	Forward Sequence	Reverse Sequence
GAPDH	CAACGGATTTGGTCGTATT	GATGGCAACAATATCCACTT
IGF1R	ACCCGGAGTACTTCAGCGC	CACAGAAGCTTCGTTGAGAA
IL1β	TTACAGTGGCAATGAGGATGAC	TGTAGTGGTGGTCGGAGATTC
IL18	GATAGCCAGCCTAGAGGTATGG	CCTTGATGTTATCAGGAGGATTCA
ASC	CTCCTCAGTCGGCAGCCAAG	ACAGAGCATCCAGCAGCCAC
NLRP3	TGGCTGTAACATTCGGAGATTG	GAAGTCACCGAGGGCGTTGT
Caspase 1	TGCCTGTTCCTGTGATGTGG	TGTCCTGGGAAGAGGTAGAAACATC
Caspase 4	AAGAGAAGCAACGTATGGCAGGAC	GGACAAAGCTTGAGGGCATCTGTA
Caspase 5	GGTGAAAAACATGGGGAACTC	TGAAGAACAGAAAGCAATGAAGT
IL33	CAC CCC TCA AAT GAA TCA GG	GGA GCT CCA CAG AGT GTT CC
IGFIEa	GTGGAGACAGGGGCTTTTATTTC	CTTGTTTCCTGCACTCCCTCTACT
IGFIEb	ATGTCCTCCTCGCATCTCT	CCTCCTTCTGTTCCCCTC
IGFIEc	CGAAGTCTCAGAGAAGGAAAGG	ACAGGTAACTCGTGCAGAGC
IGFBP3	TCCAGGAAATGCTAGTGAGTCGGA G	CTT GCT CTG CAT GCT GTA GCA GTG C
IGFBP6	GCAGAGGAGAATCCTCCTAAGGAGAG	CAGCCAACACCAACACTCTTTC

Supplementary Table S2. Demographic, clinical and laboratory features of SS patients according to lymphoma status.

	SS (n=192)	SS-lymphoma (n=77)	HC (n=337)	p-value
**Age at study entry (mean±SD)**	59.7±10.5	61.8±9.6	55.5±9.0	ns
**Female sex (%)**	179 (93.2)	69 (89.6)	30 (93.8)	ns
**Age of onset (mean±SD)**	52.5±12.9	51.7±13.8	na	ns
**Dry mouth (%)**	92.4	98.7	na	ns
**Dry eyes (%)**	86.3	94.8	na	ns
**Abnormal Schirmer's test (%)**	77.8	93.8	na	ns
**Rose Bengal Stain (≥4/9) (%)**	62.7	46.2	na	ns
**Antinuclear Antibodies ≥1/320 (%)**	85.1	94.7	na	**0.03**
**Antibodies against Ro/SSA (%)**	75	84.4	na	ns
**Antibodies against La/SSB (%)**	36.5	49.4	na	ns
**Rheumatoid Factor positivity (>20 IU/ml) (%)**	51.4	76.7	na	**≤0.001**
**Erythrocyte sedimentation rate (mm/h) (mean±SD)**	30.6±23.3	36.4 ±30.5	na	ns
**White Blood cell count (absolute number) (mean±SD)**	5726 ±2105	5342±1839	na	ns
**Neutrophil Number (absolute number) (mean±SD)**	3386±1712	3321±1705	na	ns
**Monocyte Number (absolute number) (mean±SD)**	398±192	396±193	na	ns
**Lymphocyte Number (absolute number) (mean±SD)**	1719±735	1454±608	na	ns
**C4 (mg/mL) (mean±SD)**	20.21±9	14.7±7.7	na	ns
**C3 (mg/mL) (mean±SD)**	98.96±25.3	114.9±68.8	na	ns
**IgG mg/dl (mean± SD)**	1597.4±672.6	1881.997.2	na	ns
**IgM mg/dl (mean± SD)**	150.5±117.5	206.7±219.2	na	ns
**LDH IU/L (mean± SD)**	234.3±94.5	253.3±102.9	na	ns
**Focus score (number of foci/4mm^2) (mean±SD)**	1.8±1.8	3.6±2.9	na	**≤0.001**
**Tarpley score (mean±SD)**	1.9±1.0	3.3±0.6	na	ns
**Salivary gland enlargement, n (%)**	23.3	60.5	na	ns
**Arthralgias/Myalgias, n (%)**	67.3	61	na	ns
**HGB (g/dl) (mean±SD)**	13.3±4.2	14.2±1.5	na	ns
**Raynaud's phenomenon, n (%)**	20.6	32.9	na	**≤0.001**
**Palpable purpura, n (%)**	8.2	33.8	na	ns
**Monoclonal gammopathy, n (%)**	6.4	20.8	na	**0.01**
**Lymphadenopathy, n (%)**	11.8	33.8	na	**≤0.001**
**PNS vasculitis, n (%)**	2.1	9.1	na	0.08

PNS vasculitis: peripheral nervus system vasculitis; HGB: hemoglobin; LDH: lactate dehydrogenase; ns: not significant

Supplementary figures

Supplementary Figure. S1. IGF1/IGF1R axis components mRNA expression (measured by Real-Time PCR), in MSG tissues derived from SS patients and SC.

A. IGF1R transcript levels did not differ between SS patients and SC (0.9 ±1 vs 1±1.4, p=0.55) peripheral blood.

B. IGFIEa transcript levels were higher but not significantly different in SS patients compared to SC (2.9 ±5.4 vs 1.5±1.1, p= 0.48).

C. IGFIEb transcript levels were lower but not significantly different in SS patients compared to SC (3±3.3 vs 5.6±9.5, p=0.67).

D. IGFIEc transcript levels were lower but not significantly different in SS patients compared to SC (1.6±4.3 vs 2.1±3.8, p=0.87).

E. IGFBP3 transcript levels were higher but not significantly different in SS patients compared to SC (1.4±2.3 vs 0.9± 1.6, p=0.4).

F. IGFBP6 transcript levels were higher but not significantly different in SS patients compared to SC (2.3±2.1 vs 1.6±0.7, p=0.8).

SS: Sjögren's syndrome; SC: Sicca controls; HC: healthy controls; MSG: minor salivary gland, $p \leq 0.05$

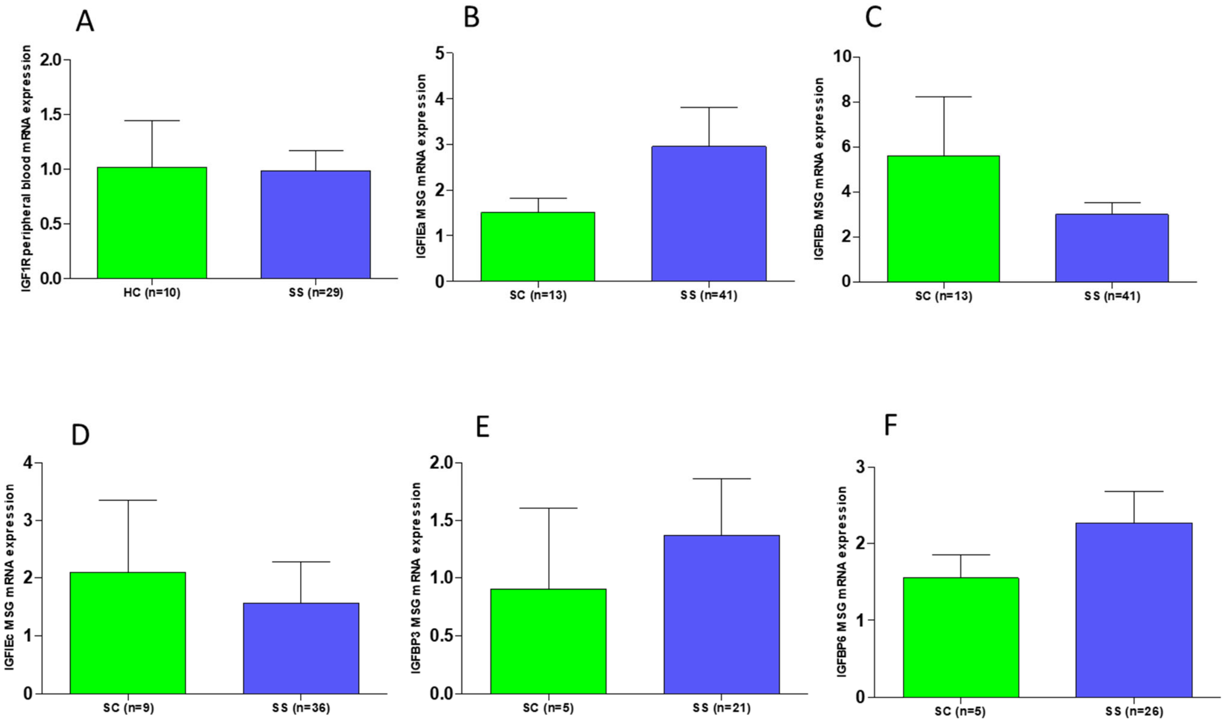

Supplementary Figure S2. Inflammatory apoptosis components mRNA expression (measured by Real-Time PCR) in MSG tissues derived from SS patients compared to SC.

A. Caspase 1 transcript levels were significantly increased in SS patients compared to SC (0.8± 0.7vs 0.3±0.1, p=0.012).

B. NLRP3 gene expression was significantly higher in SS patients compared to SC (0.2±0.2 vs 1.1± 2.2, p-value: 0.02).

C. ASC gene expression was significantly higher between SS and SC (3.3±1.9 vs 9.6± 6.6, p=0.001, p-value: 0.001).

D. Caspase 4 transcript levels were significantly increased in SS patients compared to SC (0.3±0.2 vs 1.1±1.6, p=0.03).

E. Caspase 5 transcript levels were significantly increased in SS patients compared to SC (0.2±0.1 vs 1.7± 2.4, p=0.007).

F. IL33 gene expression was significantly increased in SS patients compared to SC (6.0±7.1 vs 1.6±0.6, p=0.025)

G. Higher but not significant IL1β gene expression between SS and SC (0.5±0.7 vs 4.0± 5.6, p-value > 0.05).

H. Higher but not significant IL18 gene expression between SS and SC (4.3±3.1 vs 22.5± 44.5, p-value > 0.05).

SS: Sjogren's Syndrome; SC: Sicca controls; MSG: minor salivary gland, NLRP3: NLR- pyrin domain containing 3; ASC: apoptosis-associated speck-like; p<0.005

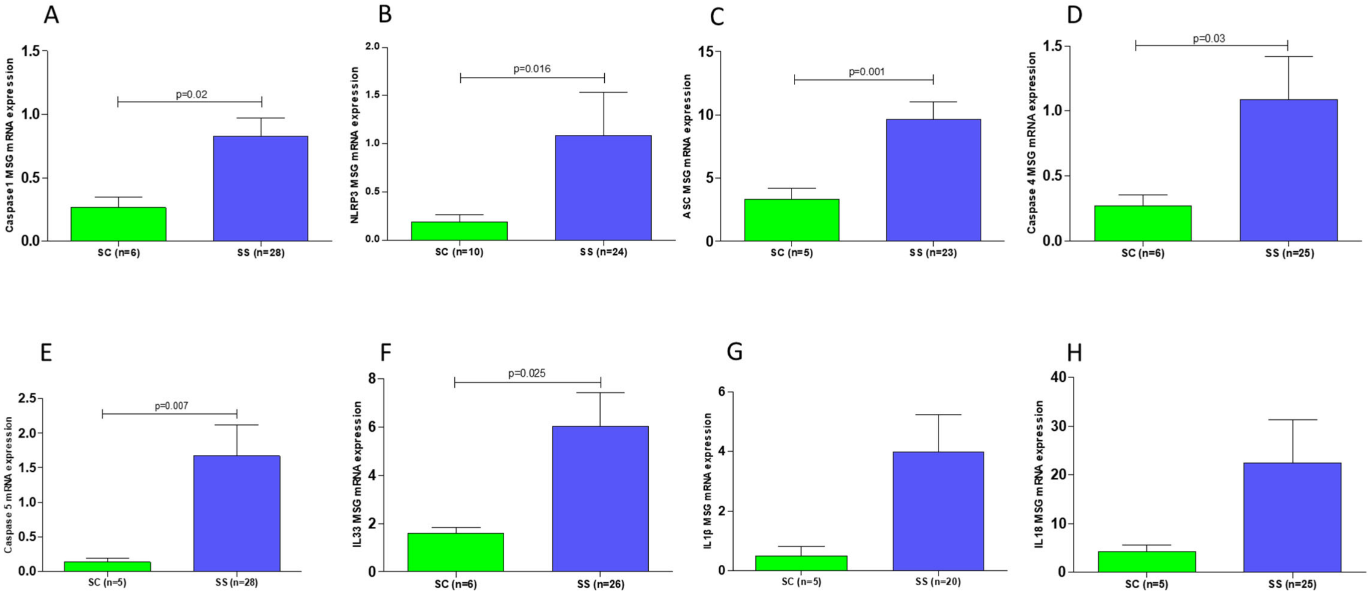

Supplement: Supplementary file 1 [file jcm-10-03960-s001.zip › jcm-1335353-supplementary.pdf]
